# Use of buccal fat pad to repair post-extraction peri-implant bone defects in the posterior maxilla. A preliminary prospective study

**DOI:** 10.4317/medoral.20212

**Published:** 2015-08-04

**Authors:** María Peñarrocha-Diago, Rocío Alonso-González, Amparo Aloy-Prósper, David Peñarrocha-Oltra, Fabio Camacho, Miguel Peñarrocha-Diago

**Affiliations:** 1 Full Professor of Oral Surgery. Stomatology Department. Faculty of Medicine and Dentistry. University of Valencia, Spain; 2 Master in Oral Surgery and Implant Dentistry, Stomatology Department. Faculty of Medicine and Dentistry. University of Valencia, Spain; 3 Master in Oral Surgery and Implant Dentistry. Collaborating Professor of Oral Surgery, Stomatology Department. Faculty of Medicine and Dentistry. University of Valencia, Spain; 4 Full Professor of Oral Surgery. Stomatology Department. Faculty of Medicine and Dentistry. University of Murcia, Spain; 5 Professor and Chairman of Oral Surgery and Implantology, Valencia. University Medical and Dental School, Valencia, Spain

## Abstract

**Background:**

Extensive literature exists about the use of the BFP in the treatment of oral defects but, to our knowledge, no article refers to the use of the BFP as a substitute of the membrane barriers for treatment of peri-implant bone defects. The aim was to evaluate the use of the buccal fat pad as a coating material for bone grafting in the peri-implant bone defect regeneration of immediate implants placed in the posterior maxilla.

**Material and Methods:**

A preliminary prospective study of patients involving immediate implants in which the buccal fat pad was used as a coating material to peri-implant bone defects was carried out. The outcome measures assessed were: postoperative pain and swelling, complications related to buccal fat pad surgery, implant survival and success rates and peri-implant marginal bone loss at 12 months of prosthetic loading.

**Results:**

Twenty-seven patients (17 women and 10 men) with a mean age of 55.3 ± 8.9 years, and a total of 43 implants were included. Two-thirds of the patients reported either no pain or only mild intensity pain and moderate inflammation, two days after surgery. Post-operative period was well tolerated by the patients and no serious complications occurred. None wound dehiscence occurred. Implant survival and success rates were 97.6% and the average marginal bone loss 1 year after loading was 0.58 ± 0.27 mm.

**Conclusions:**

Within the limits of this preliminary study, the use of the buccal fat pad as a coating material for bone grafting in peri-implant bone defects placed in the upper posterior maxilla was a well-tolerated technique by patients; high implant success rate was achieved with a minimal peri-implant marginal bone loss at 12 months of prosthetic loading.

**Key words:**Buccal fat pad, immediate implant, peri-implant bone defect.

## Introduction

Implants placed into extraction sockets have been shown to be a successfully predictable treatment modality (1). Recent reports have demonstrated clinically successful filling of the post-extraction marginal peri-implant defects using guided bone regeneration techniques (2). The successful osseous reconstruction of oral defects by bone grafting is dependent on the early physical protection of the graft from trauma and micro motion and the supply of blood to the graft (3). A recent study achieved both of these prerequisites using the buccal fat pad (BFP) as a membrane supporting the bone graft (4).

The BFP is a rounded fatty structure located between the buccinator muscle and the anterior margin of the masseter muscle and it is wrapped within a thin fascial envelope (5). Extensive literature exists about the use of the BFP in the treatment of oral defects (6). The BFP flap, especially the pedicled type, has been used most commonly for the closure of oro-antral or oro-nasal communications (7-15). The largest series were published by Poschel *et al*. (8) and Dolanmaz *et al*. (9) with 161 and 75 patients, respectively, with oroantral communications; all patients had a favorable healing course after the operation and the wounds successfully epithelized in 3-4 weeks after surgery. The second common use of the BFP has been in the closure of post excision defects due to maxillary cyts or tumor resections (16). Other minor uses have been in the closure of mucosal defects (17,18), in cleft palates (19) or in repairing the perforated sinus membrane during sinus augmentation (20,21). Other authors have published isolated clinical cases using the BFP, as a substitute of the membrane, to cover the lateral sinus wall after performing direct sinus augmentation (3,4) and to cover block bone grafts in reconstructive procedures in order to enhance immediate primary soft tissue closure and long-term soft tissue thickness, both as pedicled graft (3,22) and as free tissue graft (23). However, to our knowledge, no article refers to the use of the BFP as a substitute of the membrane barriers for treatment of peri-implant bone defects.

The aim of the study was to evaluate the use of the buccal fat pad as a coating material for bone grafting in the peri-implant bone defect regeneration of immediate dental implants placed in the posterior maxilla. This was done by assessing the postoperative pain and swelling, complications related to the fat pad technique, implant survival, implant success, and radiographic peri-implant marginal bone loss at 12 months of prosthetic loading.

## Material and Methods

The present study is reported in accordance with the STROBE statement for strengthening the reporting of observational studies (24).

- Patient Selection

We conducted a preliminary prospective clinical study involving patients with peri-implant buccal bone defects after receiving immediate implant placement in the posterior maxilla (molar zone), treated with simultaneous particulate bone grafting and were the buccal fat pad was employed as a coating material. Patients were given full information about the surgical procedures and treatment alternatives and duly signed informed consent forms. Preoperative analysis included complete medical histories and clinical and radiographic examinations with panoramic radiographs. The inclusion of patients was determined during surgery depending on the difficulty of sealing soft tissue and the existence of post-extraction bone defects, which could not be predicted prior to surgery. Patient and site inclusion and exclusion criteria are detailed in  Table 1.

**Table 1:**
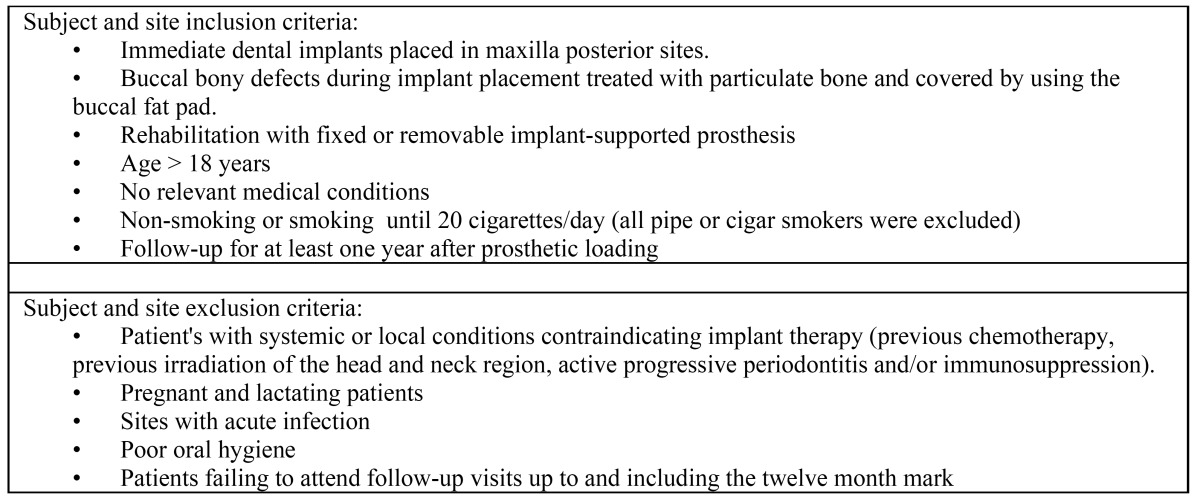
Inclusion and exclusion criteria.

This research was performed following guidelines established by the Declaration of Helsinki for human research. In this way, all patients were provided with information about the study and procedures and were asked to sign a written informed consent form before taking part. The study design was approved by the University of Valencia ethical board (Ref. H1347910943099).

- Surgical technique 

**Figure 1:**
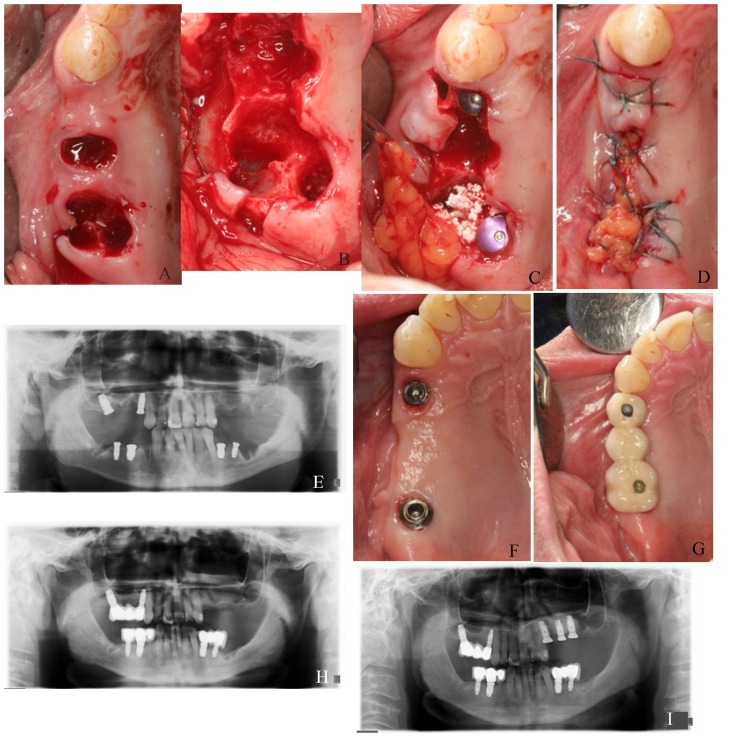
A) Post-extraction sockets corresponding to 1.5 and 1.6 positions. B) Post-extraction alveolar bone defects visualized after flap elevation. C) Dental implants placement. Buccal fat pad is placed by covering particulate bone graft. D) Suture. Buccal fat pad is left exposed to the oral environment. E) Panoramic radiography taken at dental implants placement. F) Healed soft tissues. G) Final prosthesis placement. H) Panoramic radiography taken at prosthesis placement. I) Twelve-month control panoramic radiograph.

**Figure 2:**
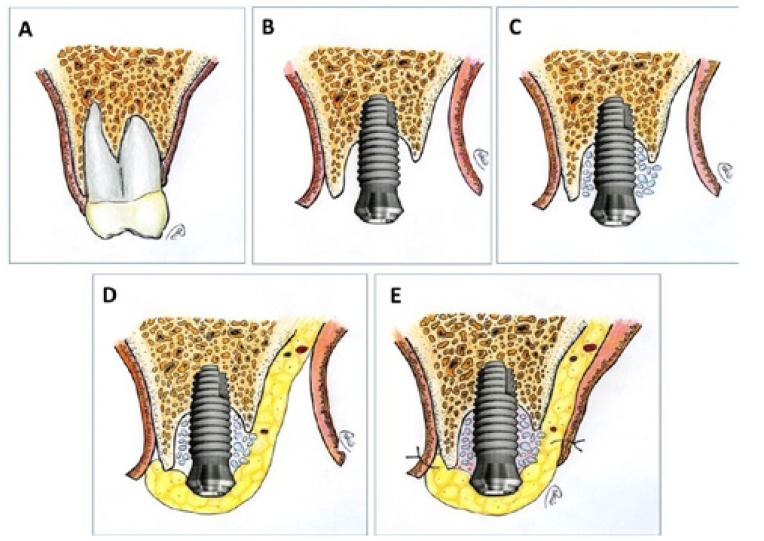
Surgical technique scheme. Sagittal view. A) Molar to be extracted. B) Immediate implant placed. C) Placement of bone graft over peri-implant defect. D) BFP buccal extension is pulled and placed over the bone graft. E) Mucoperiosteal flap replacement over buccal fat pad pedicle. Suture.

All patients received a three-drug technique for intravenous conscious sedation, administered by an anesthetist, by using the combination of midazolam, opiates and propofol at dose adapted to the needs of each patient. All procedures were also performed under local anesthesia using 4% articaine 1:100,000 adrenalin (Inibsa, Lliça de Vall, Spain). After extraction of the teeth an initial incision was made slightly palatal of the alveolar crest. One or two releasing incisions were made and a mucoperiosteal flap was raised. The exposed alveolar bone was curetted to remove all soft tissues. TSATM implants with Avantblast surface (Phibo Dental Solutions S.L., Sentmenat, Barcelona, Spain) were inserted using standard procedures following the manufacturer’s guidelines. These implants have a polished surface portion of 1.5 mm. All implants were placed with adequate primary stability (≥35 Ncm). Autologous bone graft harvested from the conformation of implant beds during drilling was adjusted to the bone contour; when the autologous bone obtained was of insufficient quantity to cover the peri-implant defects, synthetic bone (Kera-OsTM, Keramat, Coruña, Spain) was added. Buccal fat pad was then pediculate to cover the bone graft as a flap. To obtain the BFP flap, vertical discharges were widened to bottom of the vestibule. A horizontal 1-cm in length-incision was then made through the periostium, posterior to the area of the zygomatic buttress, at the zone of the second premolar and extending in anteroposterior. Blunt tip scissor was introduced directing it to the temporo-mandibular angle to separate the fibers of the buccinator muscle. Mechanical suction must be avoided once the BFP is exposed. It easily herniates with little teasing and is gently pulled out from its bed with a scissor tip. The required amount of BFP was pulled, placing it on the bone graft. For its attachment/immobilization, BFP was fixed to the palatal mucosa by a simple non-re sorbable suture. Finally, mucoperiostical flap was recovered to its original position and tension-free closures were attached with horizontal sutures using Polisoft® 4/0 sutures (Sweden & Martina, Due Carrare, Italy). A part of the fat pedicle was left exposed to the oral cavity (Fig. 1A-I ) (Fig. 2 A-E).

- Postoperative care 

Amoxicillin 500 mg and Ibuprofen 600 mg were prescribed to be taken three times daily for 7 days. Patients were also instructed to rinse with 0.12% chlorhexidine digluconate three times daily for two weeks following bone grafting and implant placement surgeries. Patients were not allowed to use removable prostheses for three weeks after bone grafting surgeries. A soft diet was recommended for one week and patients were instructed to avoid brushing or any other trauma to the surgical sites. Sutures were removed one week after surgery. Second surgeries were performed two or three months after implant placement and final prostheses were placed one month later.

- Data collection and follow-up

All data collection was carried out by a single trained clinician, different from the surgeons or the prosthodontist, following a pre-established protocol. All patients were included in a maintenance program involving annual examinations and professional prophylaxis.

Patient age (at implant placement), gender, hygiene (25) and smoking habits (none / <10 cigarettes per day / 10-20 cigarettes per day) were registered. Cause of the maxillary molars extraction (periodontitis, fracture, restorative reasons) was registered. The dimension of the defect (from the most apical aspect of the buccal crestal bone to the implant platform margin -height-, the widest mesio-distal dimension of the buccal bony defect -length-, and the distance between the implant surface and the vestibular bone margin -width-) was measured using a millimetric periodontal probe (Hu-Friedy UNC, Chicago, IL, USA). The following data were recorded: the type of the graft (autologous, synthetic bone or a mixed of both); definitive prosthesis design (single, partial or complete - fixed or over denture); type of prosthesis (cemented or screwed); implant failure (yes or no) and time of failure (months since surgery).

All patients were included in a maintenance program involving annual examinations. The following outcome measures were recorded:

Pain and swelling: Pain and swelling were recorded personally in writing by each patient at 2, 6, and 12 hours after surgery, and each day during the first 7 postoperative days. Pain intensity was assessed using a 10-cm visual analogue scale (VAS) from 0 to 10 (0 no pain, to 10 severe pain). Numerically, it was subdivided as follow: 0-2.5 as none pain, 2.6-5.0 as mild pain, 5.1- 7.0 as moderate pain and 7.1-10 as severe pain. The patients were also asked to mark the degree of their pain on a graphic rating scale. For the swelling, a subjective evaluation scale from 0 to 10 was used and subdivided in 4 parameters: none (no swelling), light (intraoral, localized to the treated area), moderate (extra oral swelling localized to the treated area), and severe (extra oral swelling extending beyond the treated area) (26).

Receptor site healing: Presence / absence of postoperative complications from the BFP manipulation were evaluated: bleeding, hematoma, wound dehiscence, local infection, partial flap necrosis, excessive granulation tissue or vestibular obliteration.

Implant survival: The criteria for implant failure were implant mobility or the removal of stable implants due to progressive peri-implant marginal bone loss or infection.

Implant success: The definition of implant success was based on the clinical and radiographic criteria put forward by Buser *et al*. (27).

Radiographic peri-implant marginal bone loss: Panoramic radiographs were made after surgery (baseline) and at the 1-year control visit to measure the peri-implant marginal bone loss. The vertical distance from the outer edge of the implant shoulder (reference point) to the most coronal point of bone-to-implant contact was evaluated at the mesial and distal aspect of each implant to the nearest 0.1 mm. Peri-implant marginal bone resorption at 1-year post-loading was calculated from the change in bone level between the baseline and the 1-year control radiograph; for each pair of measurements (mesial and distal) the largest value was used. The distance from the implant-abutment connection to the peri-implant marginal bone level was measured to the nearest 0.5 mm mesially and distally (28). Evaluation of the marginal bone level around implants was performed using image analysis software (CliniView ® version 5.1 (Instrumentarium Imaging, Tuusula, Finland). Each image was calibrated using the known length of the implants. Intra-examiner calibration was analyzed before evaluating the entire implant sample by reassessing bone loss at a total of 20 randomly selected sites (using the random function of Microsoft Excel 2010) on duplicate measurements performed on different days. An intraclass correlation coefficient of 0.904 was obtained, showing a high concordance between the two sets of data. According to Dahlberg’s d-value, a 0.052 mm error was estimated for the measurement method.

- Statistical Analysis 

Statistical analysis was performed using SPSS 17.0 software (SPSS Inc. Chicago, IL, U.S.A.). A descriptive analysis of the studied variables to obtain the means of central tendency and standard deviations was carried out.

## Results

A total of 29 patients with 46 immediate implants placed in the upper posterior maxilla -molar zone- involving peri-implants defects treated with particulate bone graft and the buccal fat pad were included. Two patients with a total of 3 implants were excluded due to lack of 12 months follow-up. The final study sample included 27 patients (17 women and 10 men), with a mean age of 55.3 ± 8.9 years (range 32-67) with a total of 43 implants. Oral hygiene was good in 68.9% and regular in 31.1% of the patients, following the Löe & Silness Index (25). Twenty patients were nonsmoking, 2 smoked until 10 cigarettes a day, and 3 were ex-smokers. In most cases, the reason for molar extraction was periodontitis (62.8% cases), followed by non-restorable caries (18.6%), failure of endodontic treatment (13.9%) and prosthetic reasons (3.7%). The mean dimensions of the resulting defects were: 5.45 mm (range 4, 5 and 8.5 mm) height, 5.27 mm (range 3 to 10 mm) length and 3.29 mm (range 3 to 5.5 mm) width. Regarding to bone graft material, 16 implants received synthetic bone; 10 received autologous bone particulate, and 17 a mixture of both. Eight implants received single restorations (3 cemented and 5 screwed), 21 implants received bridge restorations (11 cemented and 10 screwed) and 14 received full fixed restorations (3 cemented and 11 screwed).

- Pain and swelling

**Figure 3:**
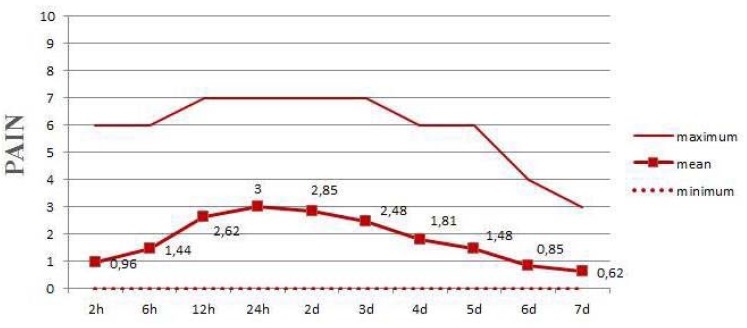
Average mean of pain levels during the first 7 postoperative days.

**Figure 4:**
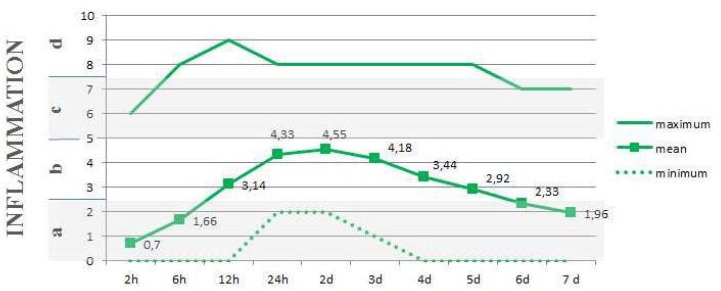
Average mean of inflammation levels during the first 7 postoperative days. Legends: a) none (no swelling); b) light (intraoral swelling, localized to the treated area); c) moderate (extraoral swelling extending beyond the treated area), d) severe (extraoral swelling extending beyond the treated area).

During first week after surgery, average of no pain to only moderate pain was reported, being the peak pain plateau recorded at 24 hours after surgery and decreasing gradually until the 7 day (Fig. 3). The swelling likewise increased progressively after surgery, reaching a maximum at the second postoperative day, at which two-thirds of the patients reported “light swelling” (intraoral swelling, localized to the treated area). It was followed by slow reduction over the subsequent days (Fig. 4).

- Receptor site healing

One patient developed extra oral swelling extending beyond the treated area accompanied by extra oral hematoma, from 12 hours until the 7th day. In any case, no serious complications occurred. The inflammation was controlled by anti-inflammatory medication.

- Implant survival, success rates, and peri-implant marginal bone loss

One implant failed after 3 months of prosthetic loading due to the lack of osseointegration. In the 42 remaining implants, there was no evidence of exudate / bleeding, peri-implant radiolucencies or clinically detectable mobility. No patients had either pain or infection. After 12-months post-loading follow-up, survival and success rates were 97.6% and mean bone loss was 0.58 ± 0.27 mm.

## Discusion

Peri-implant bone defects occurring during the surgical implant placement procedure are a frequent problem in daily practice, one with considerable clinical relevance. In order to repair these defects, guided bone regeneration has been proposed. However to ensure the success of the bone grafting procedure it is necessary to protect the bone graft material from the trauma and micro-movements and to ensure a blood supply from the recipient bed (3,4). Membrane-barries are useful to provide both of these requisites; however the risk of a membrane exposure may involve the grafting success and lead to a failure (29). The properties of the BFP have made it adequate to the reconstruction of oral defects, so, the aim of this study was to evaluate the use of the BFP as a substitute of the membrane-barrier in peri-implants defects through the outcomes related to the BFP surgery and long-term results of the implants after one year prosthetic loading.

The BFP flap has been used most commonly for the closure of oro-antral/nasal communications and mucosal defects (7-16). It also has been used to enhance immediate primary soft tissue closure at bone augmentation procedures (23). As a membrane, two authors reported the use of BFP to cover the lateral sinus wall after performing direct sinus augmentation and filling with bone graft; it was concluded that BFP might be a substitute for bioresorbable collagen membranes in maxillary and sinus floor bone grafts (20,21). BFP, as the membranes-barriers, provides an occlusive effect that will prevent connective tissue cells from colonizing the defect and at the same time, provides enough space to allow for bone regeneration of the entire defect volume (2). By placing the BFP between fast-growing fibrous tissue and the defect itself, slow-growing osseoprogenitor cells can migrate into the bone defect and lead to the reossification of this area; the use of pedicled BFP provides immediate blood supply to the recipient site and promotes rapid neovascularization of the grafted material (4). However, the BFP has other added benefits. Liversedge *et al*. (3) mentioned that the BFP has an additional protective function of providing a multilayer wound closure over all types of maxillary bone grafts, thereby preventing graft exposure and enhancing success. Furthermore, BFP pedicle over graft material may be left exposed to the oral cavity without any risk of infection since, in case of wound dehiscence, the adipose tissue allows granulation tissue formation, preventing wound sealing under tension (3). Kablan *et al*. (23) recently reported a case series of atrophic ridge bone augmentation previous implant placement in which BFP was harvesting to covering bone graft, concluding that BFP enhanced primary soft tissue closure of augmented bone and improved soft tissue thickness. However, up to our knowledge, no article refers to the use of the BFP as a substitute of the membrane barriers for treatment of peri-implant bone defects.

Most studies of postoperative pain in oral surgery have been based on the extraction of impacted teeth (26) and the placement of dental implants (30). Only two studies have involved buccal fat pad surgery (7,8), and the method to quantify these variables was only specified in one of them (7). In the present study, evaluation of pain in the first 7 days after surgery was based on a previously reported visual analogue scale (26). The visual analog scale (VAS) is considered to be a valid and reliable ratio scale for measurement of pain (31). Nezafati *et al*. (7) compared postoperative pain after BFP and mucoperiosteal flaps managements, resulting similar in both groups. In the present report a peak pain plateau was recorded starting 2 hours after surgery and persisting for 2 days; maximum pain intensity was reported at 24 hours post-surgery, decreasing gradually until the 7 day. However, it is well known that swelling perception is a highly subjective and variable experience modulated by many factors. Therefore, in this study, the level of swelling was rated in only 4 categories to simplify the rating. In relation to inflammation, most studies involving teeth extraction have reported peak swelling after 24 hours (of mild intensity). In the study published by González-Santana *et al*. (30) involving dental implants, maximum swelling was recorded after 24 hours and was of moderate intensity. In the present study, swelling peaked on the second postoperative day, when more than two-thirds of the patients presented light swelling. According to Nezafati *et al*. (7), swelling values may be expected higher in these cases since BFP flap management added a more traumatic element than other regenerative procedures. Factors such as surgical trauma, duration and surgeon’s experience directly affect the amount of swelling. However in the present study, the mean levels of swelling were light throughout the week. All procedures were performed by the same experienced surgeon under similar conditions in order to minimize this influence. The surgery was well-tolerated by all patients, similarly as reported by other authors (7,8).

The BFP management has been described in several studies as a simple and well tolerated technique, showing good results and a complete epithelialization in a few weeks (3,10). Complications of this technique are related to the management of the adipose pedicle and soft tissues. According to Baumann *et al*. (13) and Nezafati et al. (7), most complications are due to the low surgeon experience and the invasiveness of the surgical procedure itself. The most common complication was the mouth opening limitation (8,9), probably due to the fibrous scar tissue formation and shrinkage. Partial necrosis or wound dehiscence have been associated to an excessive BFP flap traction during surgery. Rapidis *et al*. (14) reported pedicle dehiscence in two patients with large maxillary defect (60 x 30 x 40 mm and 70 x 40 x 30 mm area) where tension of the BFP flap was excessive. Hassani *et al*. (4) used this technique to cover the wall after direct sinus lift and noted a decrease in the depth of the vestibule which was spontaneously resolved 2 months after surgery. In the present study a slight bleeding during the first 2 days was reported by one patient. Another patient had severe postoperative swelling, from 12 hours until the 7 day, and developed an intra and extra oral hematoma; this might be due to laceration of a blood vessel during the BFP management. To avoid these complications, Alkan *et al*. (17) recommended not to excessively tensing the adipose pedicle to avoid lacerating the pedicle and keep intact its capsule. None exposure bone graft was reported, similarly as Liversedge *et al*. (3) and Khojasteh *et al*. (22), where the primary wound closure was successful.

The present study did not include a control group with a collagen membrane and reentry was not performed, so it provides no evidence on the effectiveness of this technique on bone regeneration. Therefore, the effectiveness of the BFP was based on the morbidity, postoperative complications and dental implant survival and success rates. After 12-months prosthetic loading, radiographic data showed a minimum bone loss around the implants, and higher implant survival and success rates were reported.

Nevertheless, trials with larger sample sizes and longer follow-ups are needed to confirm or reject these findings. On the other hand, all procedures were performed by the same oral surgeon with extensive clinical experience in regenerative procedures which might limit the extrapolation of the results.

## Conclusion

Within the limits of this preliminary 1-year follow-up study, the use of the buccal fat pad as a coating material for bone grafting in peri-implant bone defects placed in the upper posterior maxilla was a well-tolerated technique by patients, and which high implant success rates were achieved, with minimal peri-implant marginal bone loss at 12 months of prosthetic loading.
